# Abnormal cerebellum connectivity patterns related to motor subtypes of Parkinson’s disease

**DOI:** 10.1007/s00702-023-02606-9

**Published:** 2023-03-01

**Authors:** Zhenzhen Chen, Chentao He, Piao Zhang, Xin Cai, Wenlin Huang, Xi Chen, Mingze Xu, Lijuan Wang, Yuhu Zhang

**Affiliations:** 1 Department of Neurology, Guangdong Neuroscience Institute, Guangdong Provincial People’s Hospital, Guangdong Academy of Medical Sciences, Southern Medical University, No. 106 Zhongshan Er Road, Guangzhou, 510080 Guangdong Province China; 2 Department of Neurology, The Central Hospital of Wuhan, Tongji Medical College, Huazhong University of Science and Technology, Wuhan, 430014 China; 3 Guangzhou Key Laboratory of Diagnosis and Treatment for Neurodegenerative Diseases, Guangdong Provincial People’s Hospital, Guangdong Academy of Medical Sciences, Guangzhou, 510080 China; 4 Center for MRI Research, Academy for Advanced Interdisciplinary Studies, Peking University, Beijing, 100190 China; 5 Guangdong Provincial Key Laboratory of Artificial Intelligence in Medical Image Analysis and Application, Guangdong Provincial People’s Hospital, Guangdong Academy of Medical Sciences, Guangzhou, China

**Keywords:** Cerebellar volume, Functional connectivity, Neural correlates, Parkinson’s disease subtype

## Abstract

Cerebellar dysfunction may substantially contribute to the clinical symptoms of Parkinson’s disease (PD). The role of cerebellar subregions in tremors and gait disturbances in PD remains unknown. To investigate alterations in cerebellar subregion volumes and functional connectivity (FC), as well as FC between the dentate nucleus (DN) and ventral lateral posterior nucleus (VLp) of the thalamus, which are potentially involved in different PD motor subtypes. We conducted morphometric and resting-state functional connectivity analyses in various cerebellar subregions in 22 tremor-dominant (TD)-PD and 35 postural instability gait difficulty dominant (PIGD)-PD patients and 38 sex- and age-matched healthy controls (HCs). The volume and FC alterations in various cerebellar subregions and the neural correlates of these changes with the clinical severity scores were investigated. The PIGD-PD group showed greater FC between the right motor cerebellum (CBMm) and left postcentral gyrus than the HC group, and a higher FC was associated with less severe PIGD symptoms. In contrast, the TD-PD group had decreased FC between the right DN and left VLp compared with the PIGD-PD and HC groups, and lower FC was associated with worse TD symptoms. Furthermore, the PIGD-PD group had higher FC between the left DN and left inferior temporal gyrus than the TD-PD group. Morphometric analysis revealed that the TD-PD group showed a significantly higher volume of left CBMm than the HC group. Our findings point to differential alteration patterns in cerebellar subregions and offer a new perspective on the pathophysiology of motor subtypes of PD.

**Supplementary Information:**

The online version contains supplementary material available at 10.1007/s00702-023-02606-9.

## Introduction

Parkinson’s disease (PD) is one of the most common progressive neurodegenerative disorders, characterized by bradykinesia, rigidity, resting tremors, gait disturbance, postural instability, cognitive abnormalities, and sleep disorders (Armstrong and Okun 2020). Patients with PD can be divided into tremor-dominant (TD) and postural instability/gait difficulty dominant (PIGD) subtypes based on their predominant motor symptoms (Nutt 2016). Non-motor dysfunctions and prognosis in patients with PD are heterogeneous, with the PIGD subtype being associated with rapid progression and increased risk of dementia compared to the TD subtype (Kann et al. 2020).

The main pathological hallmark of PD is the depletion of nigrostriatal dopamine neurons, which do not entirely account for motor and non-motor impairments in PD (Bostan and Strick 2018). A recent study found disynaptic projections originating from motor and non-motor domains within the subthalamic nucleus (STN) to the motor and non-motor regions of the cerebellum cortex (Bostan and Strick 2018). Furthermore, signals from the cerebellum converge into the dentate nucleus (DN), which then project to the ventral lateral posterior nucleus (VLp) of the thalamus, reaching vast areas of the neocortex, including the motor regions of the precentral and postcentral gyri, and associative regions of the prefrontal and posterior parietal cortex (Yang et al. 2019). Thus, the importance of the cerebellum is recognized not only in the coordination of gait, posture, and motor functions but also in its contributions to a variety of cognitive functions, such as executive function and working memory (Clark et al. 2021).

Given the anatomy of the cerebellum and its functional connectivity (FC) with the basal ganglia, it may play a key role in the pathophysiology of PD and account for some parkinsonian symptoms such as tremors and parkinsonian gait (Wu and Hallett 2013). Several neuroimaging studies have reported that distinct activities in cerebellar subregions specifically influence the motor and non-motor symptoms of PD (Wu and Hallett 2013; Maiti et al. 2020, 2021; Clark et al. 2021; Riou et al. 2021). Maiti et al. conducted morphometric and resting-state (RS) FC analyses to investigate changes in cerebellar FC in patients with PD and reported that abnormal FC in the cerebellar vermis was related to cognitive and gait impairment, respectively (Maiti et al. 2020, 2021). Another study found that a combination of cerebellar atrophy and decreased cerebellum-sensorimotor connectivity would impact the severity of patients’ motor impairment (O'Callaghan et al. 2016). Our previous study demonstrated that iron deposition in the DN was greater in patients with PD-TD than in those with PD-PIGD and healthy controls (HCs). Moreover, DN susceptibility values were correlated with tremor scores. Meanwhile, it was proposed that PD tremor may be related to the decoupling of DN from VLp (van den Berg and Helmich 2021). Overall, these findings suggest that the role of particular cerebellar subregions and DN inputs to the VLp may differ between TD and PIGD subtypes and are closely related to tremor and gait impairments, respectively. However, the precise role of the functionally subdivided cerebellar regions between patients with TD-PD and PIGD-PD has not been investigated in detail, thus warranting a direct comparison of morphological and functional changes in these regions.

We hypothesized that motor subtypes in PD are associated with specific alterations in the cerebellar subregions, gray matter (GM) volume, and FC. This study aimed to clarify the contributions of altered volume and FC in cerebellar subregions to the clinical manifestations in patients with TD-PD and PIGD-PD. We performed voxel-based morphometry (VBM) and seed-based FC to investigate volume and FC alterations in various cerebellar subregions in patients with TD-PD and PIGD-PD and the neural correlates of these changes with motor and non-motor symptoms. Lastly, as stimulation or lesions of the VLp nucleus are optimally treated to relieve Parkinsonian resting tremors (Bostan and Strick 2018), we investigated the alterations in FC between regions of interest (ROIs) in the DN and VLp and the relationship between these FC changes and tremor performance in PD.

## Methods

### Participants

Study participants were consecutively recruited at the Department of Neurology, Guangdong Neuroscience Institute, Guangdong Provincial People’s Hospital (Guangzhou, China) from August 2018 to April 2021. We recruited 92 right-handed patients with a diagnosis of PD made by two clinical neurologists with over 10 years of experience in clinical neurology according to the Movement Disorders Society criteria (Postuma et al. 2015).

All patients were either drug-naive or off anti-Parkinsonian medications for at least 12 h prior to clinical assessment and imaging scans. The assessments were performed by expert neuropsychologists who were blinded to the clinical and magnetic resonance imaging (MRI) results. We collected data on: (1) Demographic and general clinical data: age, sex, education level, age at onset, disease duration, and levodopa equivalent daily dosage (LEDD). (2) Motor manifestations: International Parkinson and Movement Disorder Society-Unified Parkinson’s Disease Rating Scale (MDS-UPDRS) Part II; MDS-UPDRS Part III (tremor and PIGD subscores); Hoehn and Yahr (H-Y) stage score. (3) Non-motor symptom manifestations: MDS-UPDRS part I; global cognitive function was assessed using the Mini-Mental State Examination (MMSE) and the Montreal Cognitive Assessment (MoCA). In this study, and in accordance with our previous study and previously published method (Stebbins et al. 2013; Chen et al. 2020), we defined the TD-PD and PIGD-PD subtypes based on the ratio of the mean tremor score to the mean PIGD score, as previously reported (Stebbins et al. 2013).

Patients were excluded if they had: (1) Any disorder that interfered with the assessment of PD (e.g., dementia and essential tremor); (2) H-Y stage score > 4 or other causes of parkinsonism; and (3) a history of head injury, seizure, brain tumor, neurologic surgeries, or other neurologic diseases. Eight patients were excluded for not meeting inclusion criteria; eight were excluded for limited clinical data; seven were excluded based on a radiologically observed cerebrovascular disease with cortical or subcortical infarcts or white matter lesions on structural MRI evaluation by two neuroradiologists; thirteen were excluded for having the “intermediate” subtype. Finally, 57 patients (22 with TD-PD and 35 with PIGD-PD) were included in the study. In addition, 37 HCs matched for age and sex were included in the study. The study protocol was approved by the Ethics Committee of Guangdong Provincial People’s Hospital and was performed in accordance with the Helsinki Declaration of 1975. Written informed consent was obtained from all participants.

### MRI data acquisition

RS-fMRI images and high-resolution 3D T1-weighted images were acquired from all PD patients in the off-state. MRI was performed on a 3.0 T scanner (Signa Excite HD GE Healthcare, Milwaukee, WI, USA) using an 8-channel head coil. The subject was positioned supine in the scanner during scanning. Foam pads and earplugs were used to reduce noise and head motion. Participants were instructed to close their eyes, neither thinking of anything in particular nor falling asleep. The functional MRI were acquired with the following parameters: gradient-echo echo-planar imaging (GRE-EPI) sequence with a repetition time (TR)/echo time(TE) = 2000/30 ms, matrix = 64 × 64, field of view (FOV), = 240 × 240 mm^2^, slice thickness = 4 mm, interslice space = 1 mm, NEX = 1, voxel size = 3.75 mm × 3.75 mm × 4 mm, time points = 186, 30 axial slices covering the entire brain, and a total of 5580 images were acquired from each subject. Subsequently, sagittal T1-weighted anatomical images were collected for co-registration with the functional data. A fast spoiled gradient recalled echo inversion recovery (FSPGRIR) sequence was used according to the following parameters: TR/TE = 8.4/3.3 ms, matrix = 256 × 256, flip angle = 13^◦^, slice thickness = 1 mm, and voxel size = 0.94 mm × 0.94 mm × 1 mm.

### Data preprocessing

Data processing was performed using the Configurable Pipeline for Analysis of Connectomes (C-PAC, htttps://fcp-indi.github.com), which is a python-based pipeline tool involving AFNI (Cox 1996), ANTs (Tustison et al. 2014), FSL (Jenkinson et al. 2012), and custom python code. The whole analysis was accelerated and simplified through the NeuroScholar cloud platform (http://www.humanbrain.cn, Beijing Intelligent Brain Cloud, Inc.).

### Structural MRI processing and volumetric analysis

Specifically, structural processing included the following steps: (1) images were de-obliqued; (2) images were re-oriented into Right-to-Left Posterior-to-Anterior Inferior-to-Superior (RPI) orientation; (3) skull stripping was performed; (4) individual skull-stripped brains were normalized to Montreal Neurological Institute (MNI) 152 stereotactic space (1 mm isotropic) with linear and non-linear registrations; (5) the brain area was categorized into gray matter (GM), white matter, and cerebrospinal fluid; (6) individual participant tissue segmentations were constrained by tissue priors from the standard space provided with FSL. The cerebellum was defined using a validated probabilistic atlas of the human cerebellum available in the SUIT toolbox for FSL (Diedrichsen et al. 2009). Cerebellar lobular and deep cerebellar nuclei masks were created using the probabilistic atlas of Diedrichsen et al. Finally, the obtained SUIT atlas was realigned back to the native subject space, resulting in GM volumes of 13 bilateral regions of the cerebellum (lobules I–IV, V, VI, Crus I, II, VIIb, VIIIa, VIIIb, IX, X, dentate, interposed nucleus, and fastigial nucleus) and eight vermis regions. In line with previous studies, lobules V, VI, VIIb, VIIIa, and VIIIb were defined as the motor cerebellum (CBMm), and Crus I and Crus II were defined as the cognitive cerebellum (CBMc) (O'Callaghan et al. 2016). The entire cerebellar vermis was combined as vermis (Maiti et al. 2020). Gray matter values of each cerebellar subregion were extracted via the SUIT cerebellar atlas and assessed for inter-group differences. The volumes of the extracted cerebellar subregions were normalized to the total intracranial volume (TIV) across all subjects to reduce head size variability (Rong et al. 2021). To investigate group differences in normalized gray matter (GM) volumes of individual cerebellum subregions, including the bilateral CBMm, CBMc, dentate nucleus (DN), and entire vermis, ANCOVA for each subregion with Bonferroni post hoc test were performed, controlling for age, sex, and TIV.

### Functional MRI processing and analysis

**Fig. 1:**
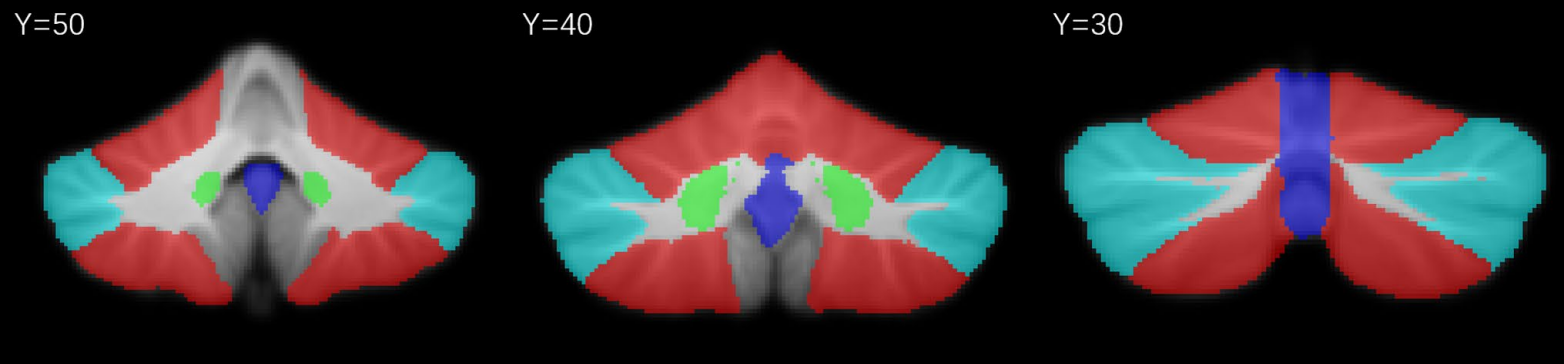
Seed regions in the cerebellar. Coronal view of the bilateral CBMm (red), CBMc (blue), dentate nucleus (green), and vermis (purple) ROIs superimposed to the spatially unbiased atlas template of the cerebellar. The CBMm was defined as bilateral lobules V, VI, VIIb, VIIIa, and VIIIb, and the CBMc was defined as the bilateral Crus I and Crus II

Specifically, functional preprocessing included the following steps: (1) the first 10 volumes were discarded to achieve complete magnet signal stabilization, and (2) slice timing correction was carried out. (3) Images were de-obliqued and re-oriented into Right-to-Left Posterior-to-Anterior Inferior-to-Superior (RPI) orientation; (4) skull stripping was performed; (5) grand mean-based intensity was normalized (all volumes scaled by a factor of 10,000); (6) functional images were registered to the anatomical space using a linear transformation, white matter boundary-based transformation, and prior white matter tissue segmentation from FSL; (7) motion artifacts were removed using ICA-AROMA with partial component regression (Pruim et al. 2015); (8) individual-level regression analysis was conducted to minimize the influence of head motion(Friston et al. 1996), signals of WM and CSF, and global signal of the whole brain; (9) all images were spatially normalized to the Montreal Neurological Institute (MNI) space and resampled as a 3 mm isotropic voxel. (10) Bandpass temporal filtering was carried out from 0.01 to 0.08 Hz. Smoothing (FWHM = 6.0 mm) was applied after Z-scoring. In addition, seven cerebellar subregions of interest were defined, bilateral “seed_CBMm,” bilateral “seed_CBMc,” bilateral “seed_DN,” and the “seed_vermis” (Fig. 1). Seed-based whole-brain functional connectivity analysis was performed using seven seeds. Each seed was correlated with all voxels to obtain whole-brain functional connectivity (FC) maps. An entire brain z-value map was created for each participant using the Fisher r-to-z transformation. Group contrasts in FC were analyzed using the bilateral CBMm, CBMc, DN, and entire vermis as seed regions.

### ROI-to-ROI analysis

Since DN is the largest deep nucleus in the cerebellum, which project to VLp to output information from the cerebellum. We performed an ROI-to-ROI analysis between the bilateral dentate nucleus and the bilateral VLp nucleus. Bilateral VLp ROIs were created using the high-resolution structural MRI-based atlas of the human thalamic nuclei (Saranathan et al. 2021). The time series for the defined ROIs were correlated to all other ROIs using the Pearson correlation. Between-group differences were tested using a one-way analysis of variance (ANOVA) with age and sex as covariates, followed by a post hoc two-sample *t* test (Bonferroni corrected).

### Statistical analysis

Statistical analyses were performed with SPSS 26.0 toolbox (version 26, IBM, New York, USA). We first evaluated the differences in demographics and clinical characteristics among the PD groups and the HC group. Continuous variables were described using means ± standard deviations (SD), while categorical variables were described using frequency counts. The Kolmogorov–Smirnov test was used to analyze the normality of the data. Comparisons between groups were performed using Fisher’s exact test for categorical variables. For continuous variables, either Student’s *t* test, one-way ANOVA (with a Bonferroni post hoc test), or Mann–Whitney *U* test, Kruskal–Wallis test were applied as appropriate, according to the normal distribution type and the homogeneity of variance of the data. For all cognitive variables, an analysis of covariance (ANCOVA) was performed for the between-groups comparisons, adjusting for age and education years. Two-tailed tests were performed, and statistical significance was set at *p* < 0.05.

Morphometric group differences between the TD-PD, PIGD-PD and HC were assessed using a univariate one-way ANOVA of the normalized volume of seven cerebellum subregions after controlling for age and sex. Bonferroni correction was used to correct for multiple comparisons in these volumetric analyses. To evaluate FC group differences, a one-way ANOVA was performed non-parametrically via Randomise tool (5000 permutations), with age and sex as covariates to map brain areas that significantly differed between groups. The ANOVA-derived map was binarized to build a mask, at *p* < 0.05 after Gaussian random field (GRF) correction. Then, between‐group two-sample *t* tests were performed within the mask showing significant differences acquired from ANCOVA analysis. The significance threshold was set at *p* < 0.001 at the voxel level and *p* < 0.05 corrected by GRF at the cluster level. To explore the relationships of FC with the clinical measures, the mean *z-*scores of the significantly altered FC between the two PD groups were extracted, and partial correlations were used after controlling for age and sex. All tests were 2-tailed, and *p* < 0.05 was considered statistically significant.

## Results

The demographic and clinical data of the 57 participants with PD and 38 HCs are summarized in Table 1. There were no significant differences in age, sex, or education between the three groups. Both PD subgroups showed lower cognitive scores than the HC after controlling for age and years of education. For patients with PD, although significant differences were noted in tremor and PIGD scores, there were no significant differences in disease duration, age of onset, LEDD, and MDS-UPDRS I-III scores between the two subgroups. However, the PIGD-PD group had a higher H-Y stage and lower cognitive score (MoCA) than the TD-PD group.

**Table 1:** Demographic and clinical data from the study groups

Variable	HC	TD-PD	PIGD-PD	*P*: HC vs TD-PD	*P*: HC vs PIGD-PD	*P*: TD-PD vs PIGD-PD
	*n* = 38	*n* = 22	*n* = 35			
Age	60.26 ± 7.18	61.27 ± 10.15	63.91 ± 8.94	1.000	0.219	0.784
Sex (male/female)	21/17	12/10	15/20	0.520		
Education (years)	11.26 ± 3.40	8.91 ± 3.37	9.96 ± 4.76	0.086	0.485	0.997
Disease duration (years)	–	2.85 ± 1.95	3.35 ± 2.90	–	–	0.771
Age of onset	–	58.50 ± 10.34	60.63 ± 8.42	–	–	0.399
LEDD	–	211.93 ± 190.80	237.86 ± 201.64	–	–	0.655
MDS-UPDRS I	–	9.95 ± 5.97	9.60 ± 5.98	–	–	0.828
MDS-UPDRS II	–	9.00 ± 5.56	9.89 ± 6.54	–	–	0.601
MDS-UPDRS III	–	31.95 ± 12.32	36.20 ± 12.12	–	–	0.206
Tremor score	–	9.45 ± 4.49	4.20 ± 3.61	–	–	< 0.001
PIGD score	–	2.23 ± 1.51	4.40 ± 2.06	–	–	< 0.001
Hoehn and Yahr	–	1.93 ± 0.44	2.39 ± 0.56	–	–	**0.001**
ADL	–	15.98 ± 4.20	18.41 ± 6.94	–	–	0.092
MMSE	28.95 ± 0.93	27.68 ± 1.94	26.71 ± 2.71	0.046	< 0.001	0.203
MoCA	26.87 ± 1.83	23.45 ± 3.88	20.71 ± 4.80	0.001	< 0.001	**0.015**
HAMA	2.79 ± 2.91	8.00 ± 5.89	10.97 ± 6.24	< 0.001	< 0.001	0.344
HAMD	2.03 ± 1.99	10.86 ± 7.66	12.94 ± 7.24	< 0.001	< 0.001	0.952

Values are reported as mean ± standard deviation and percentage frequency (%) for continuous and categorical variables, respectively

Differences between PD groups and healthy controls were assessed using *t* test, one-way ANOVA, or Kruskal–Wallis *H* test as appropriate with Bonferroni correction (for continuous variables), and Fisher test (for categorical variables)

Bold values indicate statistically significant *p* values (*P* < 0.05)

*n* number, *HC* healthy controls, *PD* Parkinson’s disease, *TD* tremor dominant, *PIGD* postural instability and gait difficulty, *LEDD* levodopa equivalent daily dose, *MDS-UPDRS* movement disorder society-sponsored revision of the unified Parkinson’s disease rating scale, *ADL* activities of daily living, *MMSE* mini-mental state examination, *MoCA* Montreal cognitive assessment, *HAMA* Hamilton anxiety scale, *HAMD* Hamilton depression scale

**Fig. 2:**
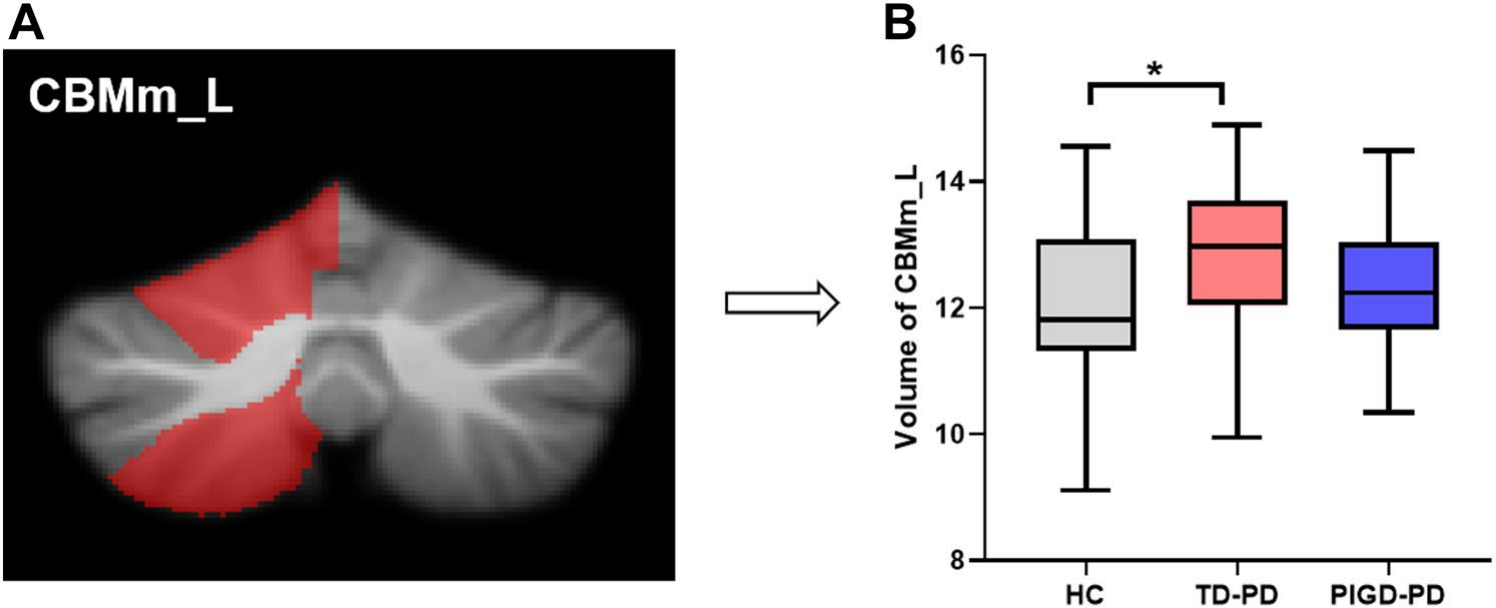
Cerebellar gray matter (GM) volumes analysis. **A** GM volumes of the left CBMm. **B** Boxplots depicts the volumetric differences among the HC, TD-PD and PIGD-PD groups. The median marks the mid-point of the data and is shown by the black line. The upper and lower whiskers represent the highest and the lowest values, respectively. Significance was set at *p* < 0.05, Bonferroni corrected

The TD-PD group showed significantly higher volumes of the left CBMm than the HC group (Fig. 2). No other significant differences were found among the three groups (Table S1 in the Supplementary Material).

**Fig. 3:**
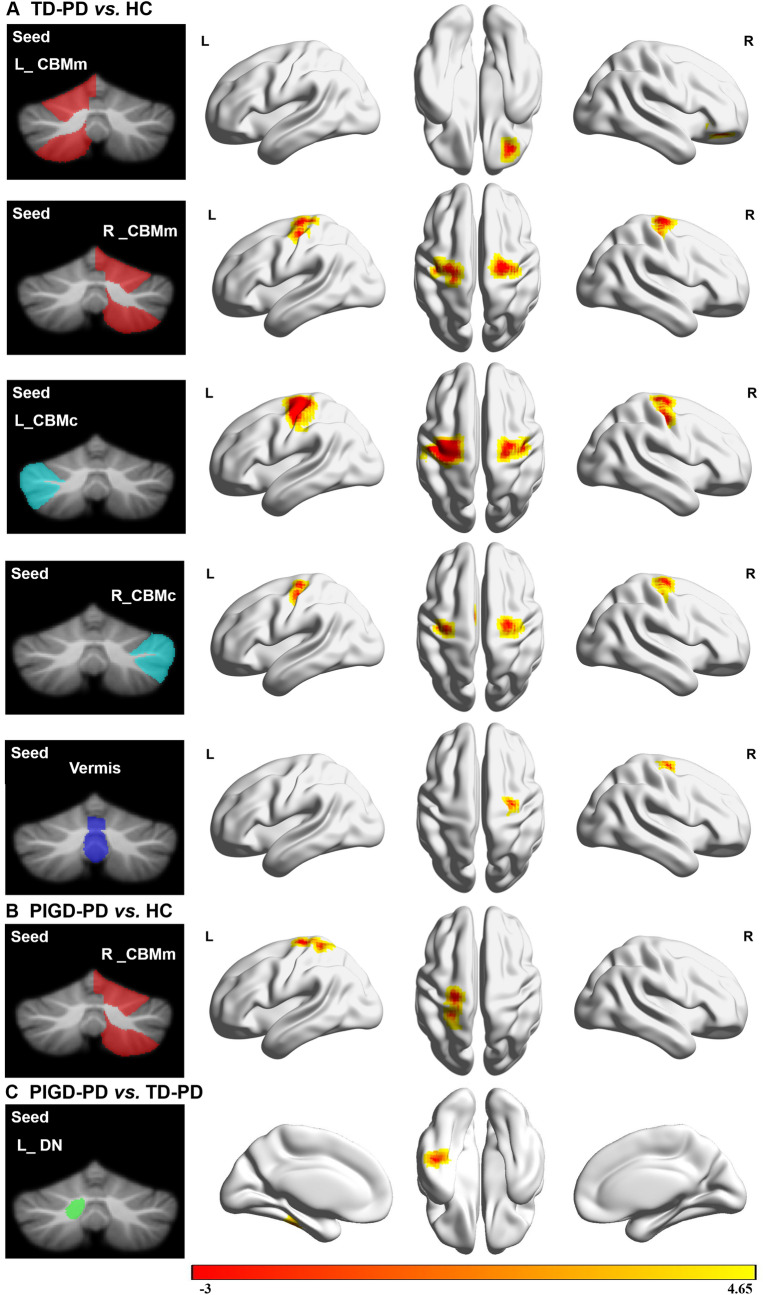
Functional connectivity (FC) differences of the cerebellar seeds between PD patients and the HC group. **A** FC (z maps) differences of seeds (bilateral CBMm andCBMc, and vermis) between the TD-PD patients and the HC group. **B** FC (z maps) differences of the seed (right CBMm) between PIGD-PD patients and the HC group. **C** FC (z maps) differences of the seed (left DN) between the PIGD-PD and TD-PD patients. Results were Cluster *p* < 0.05, Voxel *p* < 0.001, GRF corrected. Warm color represents the greater FC. *PD* Parkinson’s disease, *TD* tremor-dominant, *PIGD* postural instability and gait difficulty, *HC* healthy controls, *DN* dentate nucleus, *L* left, *R* right

In comparison with the HCs, both TD-PD and PIGD-PD groups showed higher FC, mainly between the motor cerebellum and sensorimotor areas. In detail, using the bilateral CBMm as seeds, the TD-PD group showed greater FC between the left CBMm and left middle frontal orbital gyrus (ORBmid) and between the right CBMm and bilateral precentral gyrus. Moreover, the PIGD-PD group showed greater FC between the right CBMm and left postcentral gyrus than the HC group. Using the bilateral CBMc as seeds, the TD-PD group showed greater FC between the bilateral CBMc and the bilateral precentral gyrus, left supplementary motor areas (SMA), and left middle cingulum gyrus than the HC group. Using the entire vermis as a seed, the TD-PD group also had higher FC with the right precentral gyrus than the HC group. As for the two PD subgroups, patients with PIGD-PD had significantly higher FC between the left DN and left inferior temporal gyrus (ITG) than patients with TD-PD. (Table 2 and Fig. 3).

**Table 2:** Brain regions and peak MNI coordinates of significant difference clusters in FC among the HC, TD-PD and PIGD-PD groups

Peak regions	L/R	Number of voxels	Peak MNI coordinates			*T* value
			*X*	*Y*	*Z*	
TD-PD > HC						
Seed: left CBMm						
Frontal_Mid_Orb	L	59	33	39	− 9	4.05137
Seed: right CBMm						
Precentral	L	116	− 30	− 18	69	3.95037
Precentral	R	102	24	− 18	69	3.94619
Seed: left CBMc						
Precentral	L	469	− 30	− 21	51	4.85581
Precentral	R	184	36	− 18	48	4.28935
Supp_Motor_Area	L	162	− 9	− 18	51	4.33476
Seed: right CBMc						
Precentral	R	101	24	− 21	60	4.03581
Precentral	L	86	− 36	− 18	51	4.40803
Cingulum_Mid	L	49	− 12	− 6	42	4.39193
Seed: vermis						
Precentral	R	43	24	− 18	60	4.08792
PIGD-PD > HC						
Seed: right CBMm						
Postcentral	L	82	− 24	− 24	72	4.28181
PIGD-PD > TD-PD						
Seed: left DN						
Temporal_Inf	L	48	− 48	− 27	− 21	4.64996

The *T* value indicates the peak voxel of each cluster

*MNI* Montreal neurological institute, *PD* Parkinson’s disease, *HC* healthy controls, *TD* tremor-dominant, *PIGD* postural instability and gait difficulty, *CBMm* motor cerebellum comprising bilateral lobules V, VI, VIIb, VIIIa and VIIIb, *CBMc* cognitive cerebellum comprising bilateral Crus I and Crus II, *DN* dentate nucleus, *Sup* superior, *Inf* inferior, *R* right, *L* left

**Fig. 4:**
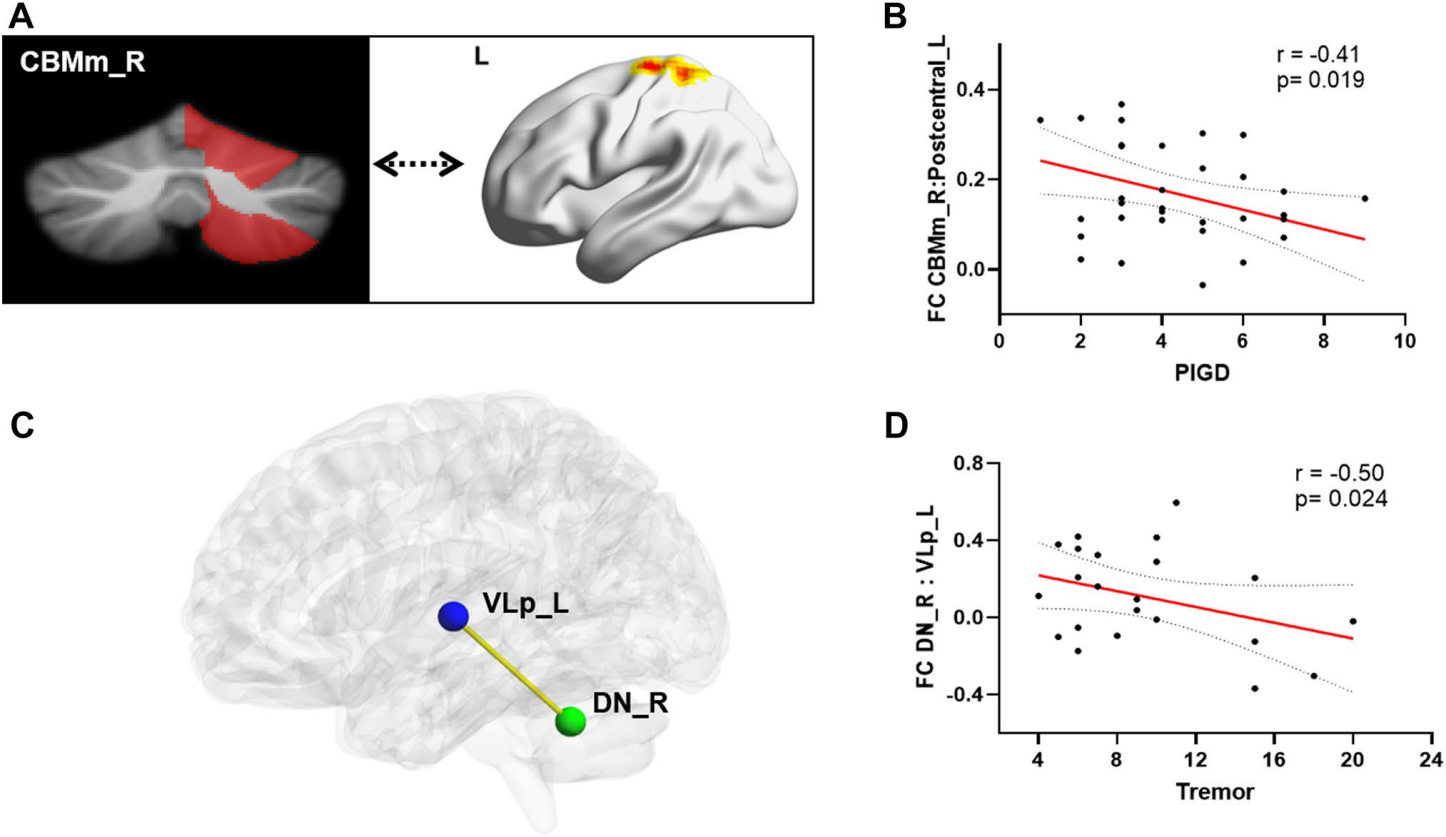
Behavioral correlation of significantly altered FC in PD participants. **A** FC between the right CBMm and left postcentral gyrus. **B** Scatterplots show correlations between the FC and PIGD scores in PIGD-PD patients. **C** FC between the right DN and left VLp. **D** Scatterplots show correlations between the FC of DN_R: VLp_L and TD scores in TD-PD patients. *PD* Parkinson’s disease, *TD* tremor-dominant, *PIGD* postural instability and gait difficulty, *HC* healthy controls, *DN* dentate nucleus, *VLp* ventral lateral posterior, *L* left, *R* right

The significant relationships of the clinical variables with the altered volume and FC values were investigated in the two PD subgroups, controlling for age and sex (Fig. 4A, B). In the PIGD-PD group, greater FC between the right CBMm and left postcentral gyrus was associated with less severe PIGD symptoms (*r* =  − 0.41, *p* = 0.019). No significant correlations were found between altered FC and the other clinical variables. The left CBMm volume was not related to any of the clinical measures.

Compared with the HC, the TD-PD group showed lower FC between the bilateral DN and left VLp, and the PIGD-PD group showed lower FC between the right DN and bilateral VLp, after controlling for age and sex (*p* < 0.05) (Table 3). To determine whether the clinical relevance of tremor and FC between DN and VLp exists, we assessed the relationship between tremor scores and FC values. Moreover, we observed that lower right DN and left VLp FC were associated with worse TD symptoms in the PD-TD group (*r* =  − 0.50, *p* = 0.024) (Fig. 4C, D).

**Table 3:** Functional connectivity differences among the HC, TD-PD and PIGD-PD groups

ROI-ROI	HC	TD-PD	PIGD-PD	*P*: HC vs TD-PD	*P*: HC vs PIGD-PD	*P*: TD-PD vs PIGD-PD
FC						
DN_L-VLp_L	0.28 ± 0.24	0.13 ± 0.20	0.14 ± 0.21	**0.039**	0.050	1.000
DN_L-VLp_R	0.27 ± 0.24	0.14 ± 0.18	0.15 ± 0.21	0.102	0.107	1.000
DN_R-VLp_L	0.29 ± 0.24	0.11 ± 0.25	0.12 ± 0.25	**0.029**	**0.016**	1.000
DN_R-VLp_R	0.27 ± 0.23	0.12 ± 0.20	0.10 ± 0.23	0.054	**0.006**	1.000

Significant differences in functional connectivity (FC) between the bilateral DN and bilateral VLp are shown

Significant group differences were obtained via one-way ANOVA followed by post hoc two-sample *t* test (Bonferroni corrected), controlling for age and sex

Bold values indicate statistically significant *p* values (*P* < 0.05)

*PD* Parkinson’s disease, *HC* healthy controls, *TD* tremor-dominant, *PIGD* postural instability and gait difficulty, *ROI* region of interest, *DN* dentate nucleus, *VLp* ventral lateral posterior, *R* right, *L* left

## Discussion

The current study observed differences in the FC of cerebellar subregions between the TD-PD and PIGD-PD groups and described correlations between altered FC and clinical variables. These findings link the cerebellar subregions to subtype-specific motor symptoms. In patients with PIGD-PD, greater FC between the right CBMm and left postcentral gyrus was associated with less severe PIGD symptoms, whereas in patients with TD-PD, lower FC of the right and left VLp FC were associated with worse TD symptoms. We propose that changes in cerebellum-based networks in specific motor subtypes of PD drive the clinical correlations between cerebellar subregion FC and motor (TD/PIGD) symptoms.

The “motor cerebellum,” consisting of lobules V, VI, VIIb, VIIIa, and VIIIb, is mostly involved in overt motor and sensorimotor processing and simple motor movements (Balsters et al. 2014). The cerebellar regulation of motor movements reveals a tightly regulated relationship between the initiation of movement via the motor cortex and cerebellum (Samson and Claassen 2017). Previous studies have suggested that the cerebellum is hyper-activated at rest, while during the execution and planning of motor tasks, the elevated activation of the cortico-cerebellum motor circuitry may play an important role in compensating for the dysfunction of the basal ganglia (Wu and Hallett 2013). Hou et al. found that both patients with TD-PD and PIGD-PD had higher FC between the cerebellum, paracentral lobule, and sensorimotor areas (Hou et al. 2018). In accordance with previous studies, we found that both patients with TD-PD and PIGD-PD had higher FC between the CBMm and sensorimotor areas than HC. Moreover, greater FC between the right CBMm and the left postcentral gyrus in patients with PIGD was associated with alleviated PIGD symptoms. One might speculate that the observed hyperconnectivity and patterns are likely to be compensated in PD, although a longitudinal study is warranted to further examine the differences in FC and progression.

Although some studies have reported cerebellar gray matter loss in PD (O'Callaghan et al. 2016), previous voxel-based morphometric analyses also found no atrophy (Maiti et al. 2020). Other studies suggest that lobule V is also involved in compensatory mechanisms of the cerebellum in patients with moderate PD (Simioni et al. 2016; Gao et al. 2017). The cerebellum is noticeably devoid of the two main pathological indicators of PD, α-synuclein deposition with Lewy bodies or mitochondrial dysfunction (Maiti et al. 2020). Consistent with previous studies, patients with TD-PD in the present study had a shorter disease duration and lower H-Y stage. Hence, the increased left CBMm gray matter volume in our patients with TD-PD may reflect a compensatory mechanism. Overall, our results support the previous findings that CBMm plays a role in the motor symptoms of PD.

The “cognitive cerebellum” is predominantly composed of Crus I and Crus II, which are involved in cognitive and executive functions (Balsters et al. 2014). The cerebellum has anatomical and functional interconnections with different neocortical areas, including the premotor, prefrontal, and posterior parietal areas of the cerebral cortex, and influences motor planning and execution, as well as cognitive functions (Samson and Claassen 2017). Using fluorodeoxyglucose PET and spatial covariance analysis, Huang et al. identified a PD-related cognitive pattern characterized by hypometabolism in the frontal and parietal association areas and correlates with cognitive performance (Huang et al. 2007). A similar finding was found in that the TD-PD group had greater FC between the bilateral CBMc and bilateral precentral gyrus, left SMA, and left middle cingulum gyrus than the HC group. Therefore, hypermetabolism in the cerebellum may also be a compensatory mechanism for maintaining cognitive function in PD. Previous studies have concluded that the TD-PD subtype is linked to fewer cognitive deficits than PIGD-PD, which is frequently associated with faster cognitive deterioration (Qian and Huang 2019). However, the mechanism underlying the relationship between cognitive deficits and postural instability/gait difficulty remains unclear. In our study, we also found that patients with TD-PD had higher cognitive scores than those with PIGD-PD. Therefore, we speculated that the increased FC between the bilateral CBMc and cerebral cortex might serve as a compensatory mechanism and partially explain the less severe cognitive performance in patients with PD-TD; however, further research is needed in the future.

The vermis includes several projections to the frontal (precentral motor), prefrontal, and posterior parietal cortices and plays a critical role in postural stability and cognitive function (Samson and Claassen 2017). Thus, degeneration of the vermis may contribute to postural instability and cognitive impairment in patients with PD. Consistent with this finding, Maiti et al. demonstrated that (1) PD participants had significantly altered FC between the vermis and sensorimotor cortex, which was correlated with subsequent gait impairment (Maiti et al. 2021); (2) PD participants had significantly altered FC between the vermis and association cortex, and the strength of this FC correlated with cognitive function. In our study, we also found abnormal FC between the vermis and sensorimotor cortex in patients with TD-PD but no correlation with clinical symptoms. The mild degree of gait and cognitive impairment in our patients with TD-PD could account for this finding. However, whether the hyperconnectivity patterns in patients with TD-PD will likely be compensated for specific pathological activity remains unclear. Longitudinal data and multimodal brain imaging studies are required to confirm this.

The DN is the main cerebellar output and projects its efferent fibers to the VLp of the thalamus and then to various motor and association cortical areas (Bernard et al. 2014). Using the DN as a seed, we observed significantly greater FC between the left DN and left ITG in the PIGD-PD group than in the TD-PD group. The ITG is known to be involved in high-order cognitive functions, including visual and language comprehension and emotion regulation (Lin et al. 2020). A previous study found that altered DN-ITG FC might reflect poor insight levels (Tikoo et al. 2021). Hence, we speculated that the altered FC between the DN and ITG in our study might reflect the pathophysiology and perhaps serve a key role in underlying cognitive impairment in patients with PIGD-PD.

Furthermore, using the ROI-ROI analysis, both the TD-PD and PIGD-PD groups showed a wide range of FC reduction between the DN and VLp groups compared to the HC group after controlling for age and sex. Using quantitative susceptibility mapping to investigate iron deposition in the deep gray matter in patients with different PD subtypes, our previous study found that DN susceptibility values were greater in the PD-TD group than in the PD-PIGD group and HC, which was correlated with tremor scores (Chen et al. 2020). Our ROI-based results corroborated this finding, as we observed significant correlations between FC values of the right DN and left VLp with tremor scores in the PD-TD group. Altogether, our results suggest that the decoupling of DN with VLp may play a cardinal role in the pathophysiology of PD tremors.

A precise balance between excitatory and inhibitory tones originating from the cerebellar cortex controls the output signals from the DN (O'Callaghan et al. 2016). However, the exact mechanism by which malfunction influences the cerebellum and/or its output pathways in the pathophysiology of PD remains unknown. Recent developments in simulating brain dynamics have revealed the impact of patients’ excitation/inhibition profiles on neurodegenerative diseases (Monteverdi et al. 2022). Future studies are needed to provide insights into the excitatory/inhibitory balance at the single-subject level and to open new avenues for brain modeling in PD.

The present study had several limitations. First, the small sample size may have led to uncertainty in the analysis results. Second, our functional imaging data acquisition protocol might have limited the conclusions, and high-quality data acquired using standard clinical scanners should be included in future studies. Additionally, as our sample was evaluated for clinical symptoms in the off-state, it will be important for future studies to include a comparison between these two groups of patients in the on-state, which can help understand the effect of treatment on FC. Moreover, “motor cerebellum” and “cognitive cerebellum” were selected based on previous studies of human and non-human primate cortico-cerebellar connectivity (Balsters et al. 2014), and we parcellated the cerebellum according to the SUIT atlas, which does not reflect all the roles of different cerebellar regions in motor and cognitive control. Because lobules I-IV in the SUIT atlas were integrated as a single area, FC analysis may have been contaminated by non-cerebellar signals. Therefore, future research using more detailed cerebellar parcellation and a whole-brain MRI-based multimodal approach (Palesi et al. 2015, 2017, 2021) should be used to reconstruct and investigate the functional changes in PD. However, our study investigated the precise role of the functionally subdivided cerebellar regions between patients with TD-PD and those with PIGD-PD, which represents an initial step towards better elucidating the roles of the cerebellum in PD.

In conclusion, in this study, we combined structural and functional MRI analyses to investigate alterations in cerebellar subregions that are potentially involved in different PD motor subtypes. This study demonstrated that an altered FC of the CBMm with the postcentral gyrus correlated with PIGD, and an altered FC of the DN with VLp correlated with PD tremors. Our findings point to differential alterations in cerebellar subregion involvement in the pathophysiology of TD and PIGD in patients with PD.

## Supplementary Information

Below is the link to the electronic supplementary material.

Supplementary file1 (DOCX 19 KB)

## Data Availability

The data that support the findings of this study are available from the corresponding author, upon reasonable request by qualified investigators.
