# Supplementary material for: Abnormal cerebellum connectivity patterns related to motor subtypes of Parkinson’s disease
Source: J Neural Transm (Vienna). 2023 Mar 1;130(4):549–60. doi: 10.1007/s00702-023-02606-9 (PMC10050038; doi:10.1007/s00702-023-02606-9)
Supplement: Supplementary file 1 — Supplementary file1 (DOCX 19 KB) [file 702_2023_2606_MOESM1_ESM.docx]

Table S1. Difference in cerebellar gray matter (GM) among the HC, TD-PD and PIGD-PD groups.

| Cerebellar | HC | TD-PD | PIGD-PD | *P*: HC vs | *P*: HC vs | *P*:TD-PDvs |
| --- | --- | --- | --- | --- | --- | --- |
| GM volume |  |  |  | TD-PD | PIGD-PD | PIGD-PD |
| CBMm_L | 12.06 ± 1.11 | 12.81 ± 1.18 | 12.31 ± 1.08 | **0.031** | 1.000 | 0.233 |
| CBMm_R | 12.06 ± 1.34 | 12.43 ± 1.19 | 12.30 ± 1.16 | 0.701 | 1.000 | 1.000 |
| CBMc_L | 9.18 ± 0.80 | 9.65 ± 0.99 | 9.34 ± 1.04 | 0.130 | 1.000 | 0.576 |
| CBMc_R | 8.44 ± 0.85 | 8.92 ± 1.01 | 8.53 ± 1.04 | 0.129 | 1.000 | 0.528 |
| Vermis | 2.36 ± 0.29 | 2.53 ± 0.31 | 2.44 ± 0.25 | 0.063 | 1.000 | 0.352 |
| DN_L | 0.08 ± 0.02 | 0.09 ± 0.03 | 0.08 ± 0.03 | 0.096 | 0.724 | 0.823 |
| DN_R | 0.05 ± 0.03 | 0.06 ± 0.04 | 0.05 ± 0.03 | 0.483 | 1.000 | 0.099 |

Significant higher volumes of the left CBMm in TD-PD patients than the HC group. Significant group differences obtained via one-way ANOVA followed post hoc two-sample t-test (Bonferroni corrected), controlling for age, sex, and TIV.

Abbreviations: PD, Parkinson's disease; HC, healthy controls; TD, tremor-dominant; PIGD, postural instability and gait difficulty; CBMm, motor cerebellum comprising bilateral lobules V, VI, VIIb, VIIIa and VIIIb; CBMc, cognitive cerebellum comprising bilateral Crus I and Crus II; DN, dentate nucleus; R, right; L, left.
